# Structural determinants of HIV inequities in South Africa: Policy analysis of the national strategic plan for HIV 2023–2028

**DOI:** 10.1016/j.hpopen.2025.100154

**Published:** 2025-11-19

**Authors:** Aqilah Julaihi

**Affiliations:** Warwick Medical School, University of Warwick, Coventry CV4 7AL, United Kingdom

**Keywords:** HIV inequities, Structural determinants, Health policy, National strategic plan, Health systems, Gender and health

## Abstract

**Background:**

South Africa continues to experience the world’s highest HIV burden despite major progress in antiretroviral therapy (ART) scale-up. Persistent disparities across gender, geography, and socioeconomic status reveal that structural and political inequities shape vulnerability, access, and outcomes. This paper examines inequalities in HIV outcomes and evaluates how social, economic, and political structures influence policy effectiveness despite expanded ART coverage.

**Methods:**

A document-based policy analysis was conducted using Walt and Gilson’s Policy Triangle framework. National strategic plans, government policy documents, and peer-reviewed literature published between 2015 and 2025 were identified through targeted database and grey literature searches. Documents addressing HIV policy, health system reform, or structural determinants were thematically analysed across four domains: policy content, context, actors, and processes.

**Results:**

Structural inequities in HIV incidence, access, and treatment outcomes remain despite expanded services. The 2023–2028 National Strategic Plan adopts a more equity-oriented and multisectoral approach, aligning with Universal Health Coverage and WHO behavioural insights. It includes differentiated service delivery, community-led monitoring, legal reform, and social protection integration. However, implementation is constrained by health system fragmentation, workforce shortages, donor dependency, and weak accountability. The suspension of PEPFAR funding disrupted community-based services, demonstrating the fragility of external financing. International experiences (eg, Thailand, Kenya) show that integrating HIV services within UHC and decentralising delivery can improve retention and equity when supported by domestic investment.

**Conclusions:**

Policy commitments increasingly recognise that equity requires structural transformation rather than solely biomedical solutions. Without stronger domestic investment, clearer implementation mechanisms, and alignment with broader reforms such as National Health Insurance, equity goals risk remaining aspirational. Embedding HIV services within a decentralised, accountable, and people-centred UHC framework offers a credible pathway to sustainability and health equity.

## Highlights

• HIV inequities in South Africa remain driven by structural determinants.
• Gender, geography, and SES influence disparities in HIV outcomes.
• The National Strategic Plan has a strong vision but faces implementation gaps.
• UHC integration of HIV services can reduce persistent inequalities.
• Donor dependence exposes the fragility of HIV programs’ sustainability.

## 1 Introduction

“Every minute, at least one person in the world still dies from AIDS-related illnesses- a stark reminder that medical breakthroughs alone cannot conquer social injustice.” [1]. Despite major advancements in HIV prevention and treatment, an estimated 39.9 million people remain affected by HIV/AIDS worldwide, including 38.6 million adults and 1.4 million children under the age of 15. Over half (53 %) were women and girls. While 86 % of individuals with HIV were aware of their HIV-positive status, about 5.4 million remained unaware they had the virus [2,3].

Health inequality is not merely a matter of statistics. At its core, it reflects how society and politics determine structural injustices that configure healthcare access and health outcomes. Although biological differences play a role, many health disparities stem from myriad inequities rooted in preventable systemic failures. These inequities shape disease patterns and disproportionately burden certain populations with greater illness and mortality [4]. The HIV/AIDS epidemic clearly illustrates this reality. Even with the success of modern medicine, many still perceive an HIV diagnosis as a death sentence. For millions worldwide, an HIV diagnosis carries significant social consequences, burdened heavily by stigma, discrimination, and exclusion [5]. The disconnect between scientific progress and public health outcomes highlights the persistent barriers to equitable healthcare access. Though antiretroviral therapy (ART) has revolutionized HIV treatment by effectively suppressing the virus and extending life expectancy, political inaction, economic inequality, and cultural stigma still heavily influence who receives treatment and who remains underserved [6].

Nowhere is this disparity more evident than in South Africa, which bears the highest HIV/AIDS burden globally, with approximately 8.3 million people living with the virus as of 2023 [7]. It serves as a stark case study of how these social and political determinants create profound health disparities [6]. Despite progress, the epidemic continues to pose a major public health crisis, as 42.3 million lives succumb to the disease [8,3]. It disproportionately affects marginalized communities due to persistent social stigma, economic inequalities, and inadequate political responses [9]. The epidemic in South Africa cannot be understood without reference to the structural legacies of Apartheid (1948-1994). During this period, health services were racially segregated: White South Africans had access to a relatively well-resourced health system, while the Black majority were confined to underfunded clinics in rural homelands and overcrowded townships [10]. Apartheid laws such as the Group Areas Act institutionalised spatial and social inequality, creating enduring disparities in housing, employment, education, and access to health infrastructure. These systemic disadvantages shaped patterns of vulnerability to HIV, particularly through labour migration, gender inequality, and poverty [11,12]. Race in this analysis is therefore understood as a social construct codified in Apartheid legislation, with lasting impacts on structural determinants of health. Despite the political transition in 1994, these inequities persist, continuing to influence who becomes infected with HIV and who has access to timely diagnosis and treatment.

The post-apartheid HIV response was also marked by political controversy. In the late 1990s and early 2000s, the Mbeki administration delayed the rollout of ART by questioning the scientific consensus on HIV, a policy failure that contributed to widespread preventable deaths. Civil society mobilisation, most notably the Treatment Action Campaign (TAC), successfully challenged the government, leading to a Constitutional Court ruling in 2002 that mandated the provision of prevention of mother-to-child transmission (PMTCT) services. From 2004 onward, South Africa scaled up the world’s largest ART programme, which has saved millions of lives and stabilised the epidemic. Yet deep inequities persist. Women and adolescent girls face disproportionate risk due to gender-based violence and unequal power dynamics; rural communities encounter barriers to testing and treatment access; and socio-economic divides continue to shape health outcomes [10].

South Africa’s HIV response has been organised through successive National Strategic Plans (NSPs), which have evolved to align with global HIV strategies and the Sustainable Development Goals. The most recent  NSP for HIV, TB, and STIs 2023–2028 outlines South Africa’s policy framework for achieving epidemic control, with explicit commitments to equity, prevention, and service integration. However, questions remain about how effectively the NSP addresses the structural determinants of inequities in HIV outcomes. This analysis is particularly timely as South Africa transitions toward Universal Health Coverage through the National Health Insurance reforms and faces growing uncertainty due to the recent pause in PEPFAR funding, making equity-focused policy evaluation urgent. The objective of this article is to critically analyse the NSP for HIV, TB, and STIs 2023–2028 using Walt and Gilson’s Policy Triangle framework, to assess how effectively it addresses the structural determinants of inequity in South Africa’s HIV response. Accordingly, the results are structured to first present the context of structural inequities (Section 3.1), before analysing the policy content, context, actors, and process of the national response (Section 3.2).

## 2 Methods

This paper employed a qualitative policy analysis approach, guided by Walt and Gilson’s Policy Triangle framework. The framework examines health policy through four interrelated dimensions – context, content, actors, and process, which allows for a systematic assessment of how political, historical, and structural factors shape both the framing and implementation of policy.

In analysing South Africa’s current HIV policy, reference was made to both the original policy documents and supporting analyses performed by others. The NSP for HIV, TB, and STIs 2023–2028 served as the primary unit of study, supplemented by the previous NSP (2017–2022) and the Department of Health’s White Paper on National Health Insurance (2015) [[13], [14], [15]] . A review of the published English-language literature for the period from January 2015 to May 2025 inclusive was conducted to ensure the analysis was grounded in the best available evidence.

A combination of Medical Subject Headings (MeSH) and free-text keywords was used to identify policy documents and policy analyses related to South African HIV policy in PubMed, Scopus, Web of Science, ScienceDirect, and BioMed Central (see Table 1). Relevant literature was also identified through the search engines Google and Google Scholar, and by checking the official websites of the South African National Aids Council (SANAC), the National Department of Health, the World Health Organization (WHO), and UNAIDS [[1], [2], [3], [7], [8], [13], [14]]. Institutional reports released by research councils and non-governmental organizations, such as the Human Sciences Research Council (HSRC), were also included[11]. Reference lists of the above-cited sources were checked to identify potentially relevant studies and reports not captured through database searches. Documents were included if they addressed HIV policy, health system reform, or structural determinants of HIV inequity in South Africa. National-level policy documents were prioritized, while documents unrelated to HIV or not relevant to health policy were excluded. Policy documents served as the primary unit of analysis (Table 1), while peer-reviewed literature and global reports were reviewed to provide contextual triangulation (Table 2) Table 3.Table 4.

**Table 1:** Key policy documents included in the analysis.

Category	Document	Year	Issuing body	Relevance to Analysis
National Policy Documents	National Strategic Plan for HIV, TB and STIs 2023–2028	2023	South African National AIDS Council (SANAC)	Primary unit of analysis; outlines current national response framework
	National Strategic Plan for HIV, TB and STIs 2017 – 2022	2017	SANAC	Previous plan; comparison for progress and continuity
	National Health Insurance For South Africa	2015	Department of Health, South Africa	Context for integration of HIV care into UHC framework
Institutional and Global Reports	SABSSM VI: National HIV Prevalence, Incidence, Behaviour and Communication Survey	2024	Human Sciences Research Council (HSRC)	Epidemiological evidence and equity indicators
	UNAIDS Global AIDS Update	2023–2024	UNAIDS	Global and regional trends, 95–95–95 targets
	WHO Global Health Observatory Data Repository	2023	World Health Organization (WHO)	Benchmarking equity and UHC metrics

**Table 2:** Supporting academic literature and reports reviewed for context.

Citation	Source	Year	Key contribution
Lewis et al. [16],Stanton et al. [17],Palanee-Philips et al. [18], Sileo et al. [19], Kim et al. [20], Bell et al. [10],Mabaso et al. [21], Leung Soo et al. [22]	Various	2019–2024	Empirical evidence on gender, geography, and socioeconomic inequities

**Table 3:** Thematic Coding framework based on Walt and Gilson’s policy triangle.

Framework Domain	Themes	Illustrative Examples from Data	Analytical Purpose
Context	Historical inequities; Apartheid legacy; donor dependency; health system fragmentation	References to structural barriers and socio-political history in NSP 2023–2028	To understand the environment shaping policy effectiveness
Content	NSP goals; strategic pillars; equity alignment with WHO principles	Donor dependence, gender-based inequity, rural infrastructure	To evaluate whether policy design addresses inequities
Actors	Government agencies; SANAC; NGOs; donors; community organisations	Role of PEPFAR, HSRC, civil society networks	To assess stakeholder influence and coordination
Process	Implementation mechanisms; monitoring and evaluation; accountability; resource allocation	NSP implementation and funding pathways	To identify where policy commitments break down in practice
Emergent Themes	Donor dependence, gender-based inequity, rural infrastructure	Secondary codes from cross-document synthesis	To capture new or unanticipated policy challenges

**Table 4:** Summary of findings by policy dimension.

Policy Dimension (Walt & Gilson)	Summary of Key Findings	Evidence of Inequity Addressed/ Not Addressed
Context	Persistent inequalities due to apartheid legacy and donor dependency	Partially addressed; structural inequities persist
Content	NSP articulates equity-focused goals and alignment with WHO behavioural insights	Addressed in design but weak in translation
Actors	Strong multi-sectoral involvement but overreliance on external donors	Not fully addressed; domestic capacity limited
Process	Implementation gaps due to limited accountability and funding volatility	Major gap: inequity persists in rural and gender dimensions

This study did not involve statistical testing or quantitative modelling because it is a qualitative policy analysis. Epidemiological figures extracted from UNAIDS, WHO, HSRC, and peer-reviewed studies were used descriptively to illustrate inequities rather than to conduct inferential analysis. All policy documents were read in full and coded thematically. A deductive coding frame was applied according to the four domains of the Policy Triangle (content, context, actors, process). Additional inductive codes were used to capture emerging themes such as donor dependency, gender-based violence, and rural health infrastructure.

The analysis focused on identifying how current policy frameworks address or fail to address inequities related to gender, geography, and socioeconomic status. Thematic synthesis was applied to extract key patterns relating to implementation challenges, political will, funding dependencies, and integration of services. Concepts from the WHO’s behavioural insights for health and Universal Health Coverage (UHC) were also integrated to assess the potential for policy adaptation and reform.

This analysis relied exclusively on documentary evidence, including national policy frameworks, global reports, and peer-reviewed literature, without primary data collection. As such, it reflects policy framing rather than lived implementation realities. All numerical data were reported as presented in source documents, without further statistical manipulation.

## 3 Results: Policy analysis using Walt and Gilson’s policy triangle

### 3.1 Evidence of structural inequities in HIV outcomes

South Africa remains the epicentre of the global HIV epidemic, bearing a disproportionate share of burden. In 2023, an estimated 13.9 percent of the population was living with HIV, representing nearly 20 percent of the global total [7,2,3]. This burden far exceeds global averages: the HIV incidence rate stands at 2.70 new infections per 1,000 uninfected individuals, compared to a global average of 0.17 [7]. Despite one of the world’s largest publicly funded ART programmes [14] and regional progress in reducing incidence and mortality, stark inequalities persist. These inequities are not biological in origin but reflect systemic disadvantages in gender, geography, race, and socioeconomic status, all of which shape exposure, access to care, and long-term outcomes.

Adolescent girls and young women (AGYW) aged 12–24 are disproportionately affected, accounting for 27 percent of new infections and being 2.5 times more likely to acquire HIV than their male peers [16,8,3]. Heterosexual transmission remains the primary route of infection, particularly in loosely committed relationships [18]. Unequal power dynamics, exposure to gender-based violence, and relationships with older men heighten this vulnerability. Inequitable gender norms further prevent both women and men from engaging fully in HIV care, while structural barriers such as long clinic queues, lack of privacy, and weak community support reinforce these patterns [19,17].

The burden of HIV in South Africa is unevenly distributed across provinces. KwaZulu-Natal and Mpumalanga report prevalence rates exceeding 25 percent in some districts, whereas the Western Cape records considerably lower figures [20]. Residents in rural areas face delays in diagnosis and poorer treatment outcomes due to long travel distances, weak transport infrastructure, and persistent shortages of healthcare workers [17]. These disparities reflect entrenched patterns of uneven resource allocation that continue to reinforce structural geographic inequities.

Inequalities also extend beyond incidence to encompass morbidity and overall well-being. In KwaZulu-Natal, individuals with uncontrolled HIV reported markedly lower health-related quality of life across multiple domains, including mobility, anxiety, and daily functioning. The presence of comorbidities such as hypertension and stroke further exacerbated these outcomes [17]. Untreated HIV remains a leading cause of premature mortality. According to the Thembisa 4.8 model, a key demographic tool for South Africa, the average life expectancy for adults with untreated HIV is only ∼ 12.5 years post-infection [23]. These findings illustrate enduring weaknesses in care continuity, service integration, and chronic disease management within the health system.

Black South Africans continue to bear the greatest burden of HIV, with Black African women facing the highest prevalence rates [10]. These inequities are deeply rooted in the historical legacy of apartheid, which systematically denied Black communities equitable access to education, housing, and healthcare [21]. The persistence of these patterns highlights how socio-political history continues to shape present-day vulnerability, demonstrating that the South African HIV epidemic is as much a legacy of structural injustice as it is a biomedical condition.

Socioeconomic disadvantage remains a powerful predictor of HIV risk in South Africa. Evidence from township populations reported by Leung Soo et al. [22] shows that individuals residing in informal dwellings are significantly more likely to contract HIV than those living in formal housing, even after adjusting for behavioural and demographic factors. Similarly, people without post-secondary education face substantially higher odds of HIV infection compared to their better-educated peers. Although insecure employment was not identified as a strong independent predictor, the combined effects of unstable housing and limited education demonstrate that vulnerability to HIV arises from structural disadvantage rather than individual behaviour. These findings are consistent with broader evidence that poverty, inadequate schooling, and marginal housing function as structural determinants that sustain South Africa’s epidemic. Collectively, these findings document a national HIV epidemic whose outcomes are fundamentally shaped by intersecting structural inequities.

### 3.2 Policy landscape

South Africa’s HIV policy has evolved through multiple phases shaped by political, social, and health system change. The initial period of political denial and delayed treatment roll-out in the early 200 s exacerbated the mortality burden described in Section 3.1, disproportionately impacting Black and poor communities. Subsequent policy cycles successfully scaled up ART, yet earlier NSPs were often implemented in a top-down manner, reflecting a biomedical orientation that failed to adequately address the gender and geographic determinants of vulnerability. A significant shift began with the 2017–2022 NSP, which incorporated concepts of combination prevention, and the Department of Health’s 2015 White Paper on National Health Insurance (NHI), which explicitly linked health inequities to the apartheid-era two-tiered system [[13], [15]]. These frameworks signalled a growing recognition of the need for structural transformation, yet their implementation remained slow and fragmented.

The current NSP for HIV, TB, and STIs 2023–2028 represents the most explicit paradigm shift to date. It positions equity, social justice, community leadership, and multisectoral collaboration as central to epidemic control [14]. This framing is a direct, if nascent, response to evidence of stark disparities in incidence, morbidity, and access. The plan’s commitment to differentiated service delivery, community-led monitoring, legal reform for key populations, and integration with social protection represents a clear intent to address the historical marginalization and poverty that sustain high transmission risks. However, the extent to which these commitments will translate into practice remains uncertain, particularly in the context of persistent health system fragmentation, workforce shortages, and donor dependency.

#### 3.2.1 Policy content: NSP vision and programmes

South Africa’s response to HIV is framed by the NSP for HIV, TB, and STIs 2023–2028, the country’s central policy framework for epidemic control. The NSP articulates four strategic goals: breaking down barriers to achieving health outcomes, maximising equitable and equal access to services and solutions, building resilient and integrated health systems, and fully resourcing and sustaining the response through inclusive governance that form a direct rhetorical response to the systemic disadvantage documented in section 3.1 [14]. These goals aim to address structural inequalities by embedding social justice, service integration, and community participation within national policy. The NSP also aligns with global best practice through its alignment with the World Health Organization’s six principles of behavioural insights, including people-centred design, the use of behavioural evidence, iterative development, stakeholder engagement, contextual understanding, and ethical rigour [24]. The plan recognizes the need to move beyond biomedical interventions and to incorporate strategies that address stigma, discrimination, and barriers to care.

Several programmatic initiatives are included within this policy framework to address populations that experience disproportionate vulnerability to HIV. Differentiated care models, including community-based ART delivery and mobile clinics, have been expanded to improve access in rural and under-resourced areas [14]. Interventions have been developed for key and vulnerable populations such as adolescent girls and young women, sex workers, people who inject drugs, and LGBTQ + individuals. These include legal empowerment services, distribution of pre-exposure prophylaxis (PrEP), and policy advocacy for the decriminalisation of sex work. The NSP also supports community-led monitoring and peer-led outreach models to improve retention in care. These efforts are complemented by multi-sectoral initiatives such as “Cash Plus” programmes that combine healthcare provision with social protection, food security, and mental health support, recognising the intersecting needs of people living with HIV [2,3].

Despite its progressive design, the NSP’s ambition has yet to be realised in practice, as evidenced by recent data. The SABSSM findings show persistent deficits in ART uptake, viral suppression, and continued high HIV incidence among AGYW, alongside structural risks such as gender-based violence and age-disparate relationships [11]. These ongoing disparities demonstrate that the policy framework has not yet overcome the historical legacies of apartheid-era resource allocation. Consequently, although the NSP positions UHC as a mechanism for equity, its implementation remains uneven, and longstanding geographic and racial inequities continue to limit progress.

#### 3.2.2 Policy context: Systemic barriers and implementation gaps

South Africa’s HIV response continues to operate within a health system marked by fragmented financing, uneven provincial capacity, and reliance on external donors. The country has not yet reached the 95–95–95 targets. Gaps in diagnosis, treatment initiation, and viral suppression mirror the vulnerabilities identified in Section 3.1 and are concentrated among rural populations, adolescent girls and young women, and other key populations. These patterns demonstrate that even large-scale biomedical interventions cannot overcome deeply rooted structural disadvantage. While the National Strategic Plan acknowledges gender-based violence, stigma, and healthcare worker bias as critical determinants of access, interventions have not yet been implemented at the scale required to address these systemic challenges [14]. Sustainable epidemic control will require stronger domestic financing, improved provincial capacity, and more effective integration of HIV services with broader health system reforms.

#### 3.2.3 Policy actors: Donor and civil society dynamics

The NSP’s multisectoral governance model envisions a response where responsibility is shared across government departments, the private sector, and community structures. Evidence from SABSSM reinforces the necessity of this coalition to address the structural drivers of the epidemic, such as intimate partner violence [11]. However, the influence of external actors creates a fundamental power imbalance that challenges this model. The recent suspension of funding from the United States President’s Emergency Plan for AIDS Relief (PEPFAR) highlighted the vulnerability of donor-dependent programmes. The withdrawal of resources disrupted service delivery in several high-burden provinces, weakened community-based care models, and interrupted continuity of care for thousands of beneficiaries [25]. The experience demonstrated that the sustainability of services for key populations often rests on volatile international priorities rather than domestic, constitutional commitments, directly undermining the NSP’s vision of local ownership.

The NHI framework recognises this structural vulnerability and proposes a unified, publicly funded health system as corrective [13]. Achieving this transition requires more than policy alignment; it demands a sovereign financial commitment to replace external funding with domestic investment. In this context, civil society has historically been instrumental in community mobilisation, advocacy for the rights of key populations, and accountability. Therefore, strengthening and domestically financing civil society is a critical structural intervention to ensure that the shift toward an integrated health system remains accountable to the populations most affected by HIV and insulated from external financial shocks [13,25,2,3].

#### 3.2.4 Policy process and strategic implications

The policy process for South Africa’s HIV response has been characterized by iterative, multi-stakeholder consultation. Both the NSP 2023–2028 and the NHI White Paper were developed through extensive national dialogues involving government, civil society, and development partners, establishing a consensus on equity and integration as foundational principles. However, this inclusive formulation has not guaranteed effective implementation. SABSSM data revealing persistent gaps in testing and viral suppression indicate that policy commitments have stalled before reaching routine practice [11]. The NHI reform pathway similarly highlights systemic constraints, specifically fragmented financing, uneven provincial capacity, and donor dependency [13]. These constraints prevent uniform service delivery and perpetuate the very geographic and racial inequities the policies were designed to eliminate.

Strengthening the policy process, therefore, requires bridging the gap between formulation and implementation. This can be achieved through coherent national-provincial coordination, clear accountability structures, and predictable domestic financing. The NSP’s emphasis on behavioural insights offers a critical tool for this, enabling a redesign of service delivery focused on core challenges. These include how patients navigate clinics, how information is communicated, and how community engagement is structured, all to reduce drop-off across the care cascade. Furthermore, integrating HIV services into primary healthcare under the NHI framework is essential for sustainability, though this is contingent on equipping provinces with the capacity and resources to implement these models effectively.

In conclusion, sustained progress toward epidemic control depends less on new strategies and more on executing existing ones with operational clarity, consistent funding, and robust systems. Ensuring that communities and frontline providers continue to shape this implementation is not just a matter of principle, but a practical necessity to ensure that the promise of equitable health outcomes, so clearly articulated in policy, finally becomes a lived reality for all South Africans.

## 4 Discussion

Marked disparities in HIV outcomes across South Africa remain both persistent and ethically unacceptable. These differences are not biologically determined but arise from avoidable social, economic, and political disadvantage. [[10], [22],]. A fair health system should provide equal opportunity for health and well-being; yet the present analysis shows how far the national response remains from this ideal. Achieving equity requires structural transformation that addresses the systemic determinants of unequal risk, access, and outcomes [[13], [26]] .

Policy content provides an ambitious framework but has not been translated into consistent practice. The NSP for HIV, TB, and STIs 2023 to 2028 sets out a comprehensive agenda to confront structural barriers and embed equity within service delivery [14]. The plan aligns with the World Health Organization’s behavioural insights principles, emphasising people-centred design, stakeholder engagement, and contextual understanding [24]. Its programme components include differentiated models of ART delivery, mobile outreach, community-led monitoring, and multisectoral initiatives that integrate social protection and mental health support [17,2,3]. Nevertheless, many of these measures remain unevenly implemented, and commitments to integration and equity are often expressed in policy texts without clear mechanisms for operationalisation or monitoring [27,28].

The policy context continues to be shaped by the enduring structural legacies of apartheid and the persistent influence of poverty, gender based violence, and geographic inequities [[10], [21], [22]]. Rural health systems remain underdeveloped, with inadequate transport networks and workforce shortages that delay diagnosis and treatment [29,17]. Economic instability restricts the ability to seek and sustain care, while low educational attainment increases vulnerability to infection [22]. Social norms, particularly those relating to gender and power, further reinforce vulnerability among women and adolescent girls [30,16,31]. Without deliberate alignment between health policy and broader social and economic reforms, interventions risk remaining vertical and fragmented, unable to shift the fundamental determinants of inequity [32,26].

Policy actors remain pivotal in shaping both policy design and service delivery. International donors, most notably the United States President’s Emergency Plan for AIDS Relief, have historically underpinned South Africa’s HIV response. The recent suspension of external funding exposed the fragility of this model, disrupting service delivery and undermining community-based initiatives in high-burden areas [25]. Civil society organisations have been instrumental in extending reach, advocating for marginalised populations, and holding institutions accountable [[5], [14]]. However, these groups remain financially and politically vulnerable. Greater domestic investment, sustained public–private partnerships, and stronger institutional support for community leadership are required to ensure continuity and resilience [13,2,3].

The policy process requires significant strengthening to translate formal strategies into practice. HIV services have been delivered predominantly through vertical programmes that are insufficiently responsive to the lived realities of patients [28,32]. Barriers such as service rigidity, stigma, and health worker bias impede retention in care and treatment adherence [27,[5], [19]]. Experiences from other settings demonstrate the feasibility of reform. Thailand integrated HIV care into its Universal Health Coverage scheme, improving treatment retention by extending antiretroviral refill intervals and embedding rights-based governance [[3], [26]]. Kenya implemented a decentralised model that combined community outreach, differentiated service delivery, and digital systems to strengthen accountability and equity [33]. These examples show how a robust, adaptive process can convert policy commitments into measurable improvements in population outcomes.

For South Africa, embedding HIV services within a decentralised and equitable Universal Health Coverage framework offers a credible and evidence-informed pathway to sustainability and fairness [13,26]. Doing so would strengthen domestic accountability, protect against funding volatility, and align service delivery with behavioural and structural determinants of health [[2], [14],^3^]. Moving beyond vertical and donor-dependent programmes toward an integrated and people-centred health system is essential if the ambitious vision of the NSP is to translate into enduring gains in equity and epidemic control.

## 5 Limitations

This study has several limitations. First, it focuses on one country with a highly unique historical and political context. While South Africa provides important lessons, findings may not be generalizable to other settings. Second, the analysis relies exclusively on documentary evidence, including national policy frameworks, global agency reports, and peer-reviewed literature. This approach enables a broad thematic understanding of how the NSP 2023–2028 frames HIV inequities. However, it does not capture lived experiences or frontline implementation challenges. Primary data collection, such as stakeholder interviews or ethnographic fieldwork, was not conducted and may have provided deeper insights into contextual barriers and enablers. Finally, future research should explore perspectives from service users and implementers, as well as comparative evaluations of policy integration across low- and middle-income countries, to deepen understanding of equity in HIV policy.

## 6 Conclusion

South Africa’s experience with HIV demonstrates that progress cannot be measured only by the scale of treatment programmes or the reduction of incidence at the national level. Behind these achievements remain deep and persistent inequities shaped by gender, geography, race, and socioeconomic status. Policies have often been well written but less effective in practice, revealing what McLoughlin et al. [34] terms the “know–do gap,” the disjuncture between carefully designed strategies and their uneven implementation. Bridging this divide requires more than biomedical solutions. It calls for structural change that confronts stigma, invests in neglected communities, and strengthens the capacity of health systems to deliver care equitably.

The National Strategic Plan for HIV, TB, and STIs 2023–2028 [14] represents an important attempt to confront these challenges. As with any arch, the integrity lies not merely in its design but in the contribution of each piece that sustains it. Policymakers, healthcare providers, civil society, and communities must all carry their share of the weight. If even one part of this arch falters, the structure risks collapse under the burden of the epidemic. The future of South Africa’s HIV response will therefore depend on sustained political will, inclusive participation, and accountability mechanisms that ensure promises are translated into action. Only through such collective effort can the epidemic be controlled, and only then will the vision of an AIDS-free generation move closer to reality.

## Ethics statement

This study was based entirely on the analysis of publicly available documents, including policy frameworks, national reports, and peer-reviewed literature. No primary data collection involving human participants was conducted. Therefore, ethical approval was not required. The research adhered to ethical standards for secondary analysis, ensuring accurate representation of sources and proper citation of all materials used.

## 9 Declaration of AI use

During the preparation of this work, the author used ChatGPT for language editing and for improving the clarity and organisation of the manuscript. After using this tool, the author reviewed and edited the content as needed and takes full responsibility for the content of the published article.

## CRediT authorship contribution statement

**Aqilah Julaihi:** Writing – review & editing, Writing – original draft, Visualization, Validation, Supervision, Software, Resources, Project administration, Methodology, Investigation, Funding acquisition, Formal analysis, Data curation, Conceptualization.

## Funding

This research did not receive any specific grant from funding agencies in the public, commercial, or not-for-profit sectors.

## Declaration of competing interest

The author declares that they have no known competing financial interests or personal relationships that could have appeared to influence the work reported in this paper.
